# Potential Physiological and Molecular Mechanisms Underlying Low Viability of Gynogenetic WW-Type Super-Female Sterlet (*Acipenser ruthenus*)

**DOI:** 10.3390/ijms27010207

**Published:** 2025-12-24

**Authors:** Ruoyu Wang, Yutao Li, Yining Zhang, Sihan Wang, Hongrui Che, Dingchen Cao, Zhipeng Sun, Bo Ma, Ying Zhang

**Affiliations:** 1 Key Open Laboratory of Cold Water Fish Germplasm Resources and Breeding of Heilongjiang Province, Heilongjiang River Fisheries Research Institute, Chinese Academy of Fishery Sciences, Harbin 150076, China; wangruoyu@hrfri.ac.cn (R.W.);; 2 College of Fisheries and Life Science, Dalian Ocean University, Dalian 116023, China; 3 College of Fisheries and Life Science, Shanghai Ocean University, Shanghai 201306, China

**Keywords:** gynogenesis, *Acipenser ruthenus*, transcriptomics, ferroptosis, lipid peroxidation

## Abstract

WW-type super-female broodstock are essential for all-female breeding in sturgeons under the ZZ/ZW sex-determination system, but their practical use is constrained by high mortality. This study investigates the underlying physiological and molecular mechanisms contributing to the reduced viability of WW-type super-female sterlet (*Acipenser ruthenus*) by comparing three genotypes (ZZ, ZW and WW) in terms of survival rates, oxidative stress levels, and gonadal gene expression. F_2_ gynogenetic diploid juvenile sterlet with three genotypes were reared for 100 days under controlled conditions. Survival rates were recorded, and oxidative stress markers, including SOD, CAT, MDA and GSH-Px, were measured using commercially available assay kits. Gonadal gene expression profiles were analyzed using transcriptomic analysis. The results revealed that WW-type juveniles exhibited a significantly lower survival rate (64.2%) compared to ZZ-type and ZW-type fish (both 94.2%, *p* < 0.0001). While hepatic SOD and CAT activities did not differ among genotypes, MDA and GSH-Px levels were significantly higher in WW-type fish, suggesting enhanced lipid peroxidation and an insufficient compensatory antioxidant response. Transcriptome analysis revealed 747 significantly differentially expressed genes between WW-type super-females and normal ZZ/ZW individuals (*p* < 0.05), with significant enrichment in pathways related to immune regulation, receptor activity, lipid metabolism, and ferroptosis. Notably, downregulation of arachidonic acid metabolism genes (PTGS2, PTGES, PTGDS) was observed, while ferroptosis-related genes GPX4 and SLC3A2 were upregulated, suggesting that disturbed arachidonic acid metabolism, along with lipid peroxidation and ferroptosis activation, contribute to the reduced survival of WW-type super-females. These findings provide integrative physiological and transcriptomic evidence for the mechanistic basis of poor fitness in gynogenetic WW-type super-females and offer foundational data for improving the feasibility of all-female breeding in sturgeon.

## 1. Introduction

Sturgeons are of great economic importance in global aquaculture due to their roe, which is processed into high-value caviar. However, sturgeon species possess several inherent features that limit aquaculture efficiency [1], including a long maturation cycle of 6–12 years, which makes the aquaculture process time-consuming and resource-intensive. Additionally, the lack of distinguishable sexual dimorphism during early developmental stages complicates sex identification, making it difficult to selectively breed for females, which have much higher economic value than males. As a result, mixed-sex rearing substantially reduces aquaculture efficiency. All-female breeding has therefore become a key strategy for reducing production costs, increasing caviar yield, and promoting development of the sturgeon industry. Under the ZZ/ZW sex-determination system in sturgeons [2], crossing WW-type super-females with normal ZZ males can produce 100% ZW female offspring. Generating stable and fertile WW-type super-females is essential for large-scale production of all-female populations. Sturgeons do not naturally undergo gynogenesis, so artificial induction is required to produce WW-type super-females. This process involves using methods like thermal shock or chemical treatments to induce parthenogenesis, generating diploid offspring without paternal genetic contribution.

Gynogenesis is a promising method for obtaining WW-type super-females and has been successfully applied in several acipenseriform species, including sterlet (*Acipenser ruthenus*) [3], Siberian sturgeon (*A. baerii*) [4], ship sturgeon (*A. nudiventris*) [5], shortnose sturgeon (*A. brevirostrum*) [6], and the hybrid bester (*Huso huso*♀ × *A. ruthenus*♂) [7]. These studies have provided important evidence supporting the ZZ/ZW sex-determination mechanism in sturgeons and established the theoretical basis for producing WW-type super-females. In recent years, the development of Z/W-specific molecular markers in sterlet has enabled accurate identification of WW-type super-females [8]. However, across these gynogenetic sturgeon populations, WW-type super-females have frequently been reported to exhibit poor physiological fitness and high mortality, which severely limits their practical use as broodstock. The physiological and molecular mechanisms underlying this reduced fitness remain unresolved.

WW-type super-females generated through gynogenesis lack paternal genetic contribution and may therefore experience disturbances in immune regulation and metabolic homeostasis, resulting in reduced adaptability. Studies in other fish species, such as common carp (*Cyprinus carpio*) [9], brown trout (*Salmo trutta fario*) [10], and rainbow trout (*Oncorhynchus mykiss*) [11], have shown that individuals with a uniparental genome often suffer from imperfect gene-dosage compensation and homozygous expression of deleterious recessive alleles. These factors lead to increased mortality rates and physiological deficiencies, including weakened immune responses and impaired metabolic regulation. When immune and metabolic functions are compromised, organisms struggle to maintain physiological homeostasis; in particular, reduced lipid metabolic efficiency renders membrane polyunsaturated fatty acids more vulnerable to reactive oxygen species, thereby promoting lipid peroxidation [12]. As lipid peroxidation products accumulate, membrane integrity and permeability deteriorate, causing immune cell dysfunction and tissue damage. This process may also trigger ferroptosis, a regulated form of cell death characterized by iron-dependent lipid peroxidation, which further exacerbates oxidative injury and reduces viability [13,14]. Therefore, the enhancement of ferroptosis induced by abnormal lipid metabolism may represent a key mechanism underlying the physiological vulnerability of WW-type super-females.

In summary, this study focuses on gynogenesis-derived F_2_ generation sterlet, constructing juvenile fish populations with ZZ, ZW, and WW genotypes. It systematically compares survival ability, antioxidant physiological traits, and gonadal transcriptomic differences, aiming to identify potential abnormalities in the physiology and molecular regulation of WW-type super-females. This study will provide insights into the potential mechanisms of high mortality in WW-type super-females, assess their feasibility as broodstock for all-female breeding, and offer theoretical foundations and technical references for large-scale all-female sturgeon breeding.

## 2. Results

### 2.1. Survival Rate of Juvenile Sterlet with Different Sex Genotypes

**Figure 1:**
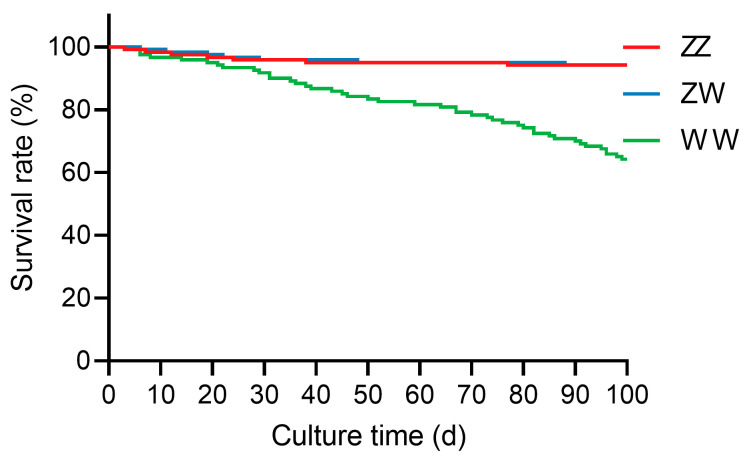
Survival rate of juvenile sterlet with different sex genotypes during 100-day culture.

During the experimental period, clear differences in survival rates were observed among the three genotypes (Figure 1). The survival rates of WW-type juveniles were significantly lower than those of ZZ-type and ZW-type individuals (*p* < 0.0001). The survival rates of the ZZ, ZW, and WW groups were 94.2% (7 deaths), 94.2% (7 deaths), and 64.2% (43 deaths), respectively.

### 2.2. Antioxidant Indices of Juvenile Sterlet with Different Sex Genotypes

**Figure 2:**
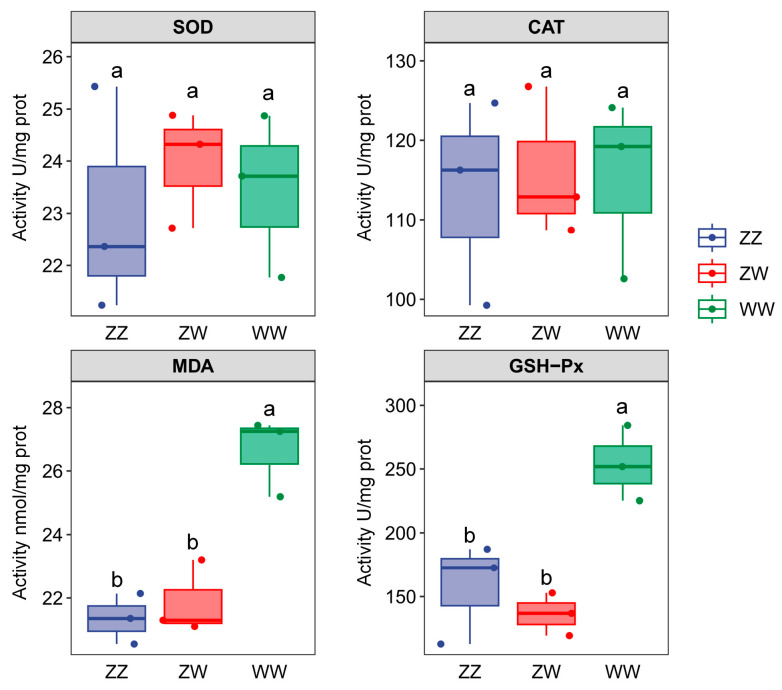
Antioxidant indices in the liver of juvenile sterlet with different sex genotypes. Different letters indicate significant difference (*p* < 0.05).

Significant differences in antioxidant capacities were observed among the different sex genotypes (Figure 2). No significant differences were detected in SOD or CAT activities among the ZZ, ZW, and WW groups (*p* > 0.05). However, MDA and GSH-Px levels in the WW-type fish were significantly higher than those in the ZZ-type and ZW-type fish (*p* < 0.05), whereas no significant differences were detected between the ZZ and ZW groups (*p* > 0.05).

### 2.3. Quality Assessment of Transcriptome Sequencing of Gonadal Tissues

The quality statistics of RNA-seq data are presented in Table 1. All samples exhibited a valid base percentage exceeding 95%, and the proportion of bases with Q30 scores was above 95%, indicating high sequencing quality. The GC content of all samples ranged from 44.07% to 46.27%, consistent with typical GC levels in fish genomes. These results demonstrate that the sequencing data were of high quality and suitable for downstream analysis.

### 2.4. Identification of Differentially Expressed Genes Between Normal Males/Females and Super-Females

**Figure 3:**
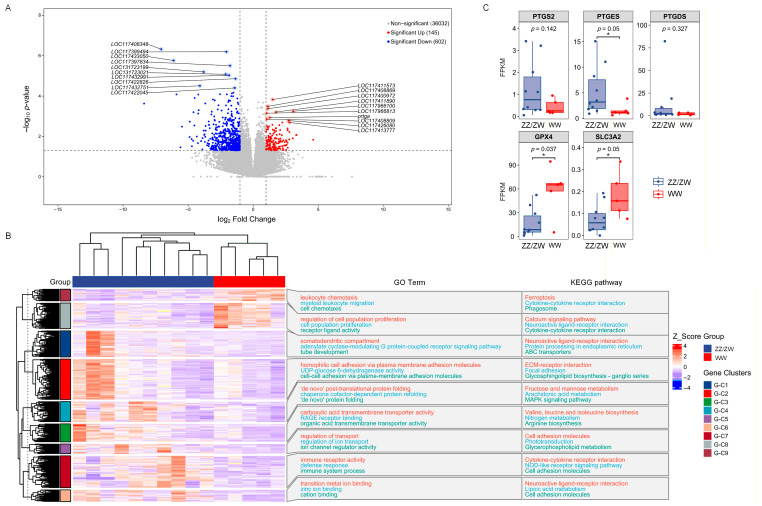
Differential gene expression analysis between WW-type super-females and normal juveniles (ZZ/ZW): (**A**) Volcano plot showing significantly upregulated and downregulated DEGs. (**B**) Heatmap of major DEG clusters with representative enriched GO terms and KEGG pathways. (**C**) Expression levels of ferroptosis-related genes (PTGS2, PTGES, PTGDS, GPX4, SLC3A2) between ZZ/ZW and WW groups. “*” indicate significant difference (*p* < 0.05).

Clear differences in gonadal gene expression patterns were observed between normal males and females (ZZ/ZW) and super-females (WW) (Figure 3). A total of 36,779 DEGs were identified between super-females and normal individuals (ZZ/ZW), of which 145 genes were significantly upregulated and 602 genes were significantly downregulated (*p* < 0.05, Figure 3A). Among the upregulated DEGs in super-females, two major gene clusters were enriched, corresponding mainly to GO terms such as leukocyte chemotaxis and regulation of cell population proliferation, and KEGG pathways such as ferroptosis and calcium signaling pathway. Among the downregulated DEGs, seven major clusters were identified, enriched in GO terms such as somatodendritic compartment, homophilic cell adhesion via plasma membrane adhesion molecules, ‘de novo’ post-translational protein folding, carboxylic acid transmembrane transporter activity, regulation of transport, immune receptor activity, and transition metal ion binding. Corresponding KEGG pathways included Neuroactive ligand-receptor interaction, ECM-receptor interaction, Fructose and mannose metabolism, Valine, leucine and isoleucine biosynthesis, Cell adhesion molecules, Cytokine-cytokine receptor interaction, and Neuroactive ligand-receptor interaction. Notably, ferroptosis-related genes showed clear expression shifts in super-females: PTGS2, PTGES, and PTGDS were significantly downregulated, whereas GPX4 and SLC3A2 were significantly upregulated (*p* < 0.05; Figure 3C).

### 2.5. GO and KEGG Enrichment Analyses of Differentially Expressed Genes

**Figure 4:**
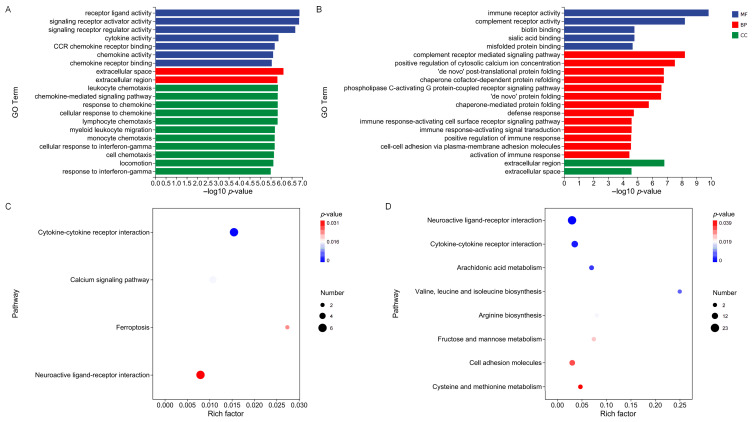
GO and KEGG enrichment analysis of DEGs between WW-type super-females and normal juveniles: (**A**) GO enrichment of upregulated genes. (**B**) GO enrichment of downregulated genes. (**C**) KEGG pathways enriched among upregulated genes. (**D**) KEGG pathways enriched among downregulated genes.

To further elucidate the biological functions and signaling pathways associated with the DEGs between normal males/females and super-females, GO and KEGG enrichment analyses were performed (Figure 4). Overall, the DEGs were mainly enriched in pathways associated with immune regulation, receptor activity, lipid metabolism, and ferroptosis. In the GO enrichment analysis, upregulated genes (Figure 4A) were mainly enriched in immune-related terms, including receptor ligand activity, signaling receptor activator activity, signaling receptor regulator activity, chemokine activity, CCR chemokine receptor binding, chemokine receptor binding, cellular response to chemokine, and leukocyte chemotaxis. Enrichment was also observed in terms related to cytokine activity and receptor binding. Downregulated genes (Figure 4B) were enriched in GO terms related to immune receptor activity, complement receptor activity, biotin binding, and chaperone cofactor-dependent protein refolding, as well as processes associated with immune response activation and cell adhesion, such as immune response-activating signal transduction and cell-cell adhesion via plasma-membrane adhesion molecules. KEGG enrichment analysis revealed that upregulated genes (Figure 4C) were primarily enriched in pathways including cytokine-cytokine receptor interaction, calcium signaling pathway, ferroptosis, and neuroactive ligand-receptor interaction. Downregulated genes (Figure 4D) were enriched in pathways related to neuroactive ligand-receptor interaction, arachidonic acid metabolism, valine, leucine and isoleucine biosynthesis, arginine biosynthesis, fructose and mannose metabolism, cell adhesion molecules, and cysteine and methionine metabolism.

## 3. Discussion

### 3.1. Significant Differences in Physiological State and Gene Expression Patterns Between Normal Male/Female and Super-Female Sterlet

Gynogenetic super-female sterlet exhibit distinct physiological characteristics compared with normal male and female individuals, a pattern commonly attributed to their uniparental genome and the absence of paternal genetic contribution [15,16]. Previous studies have shown that gynogenetic development is often associated with imperfect dosage compensation, increased homozygosity of recessive alleles, and altered developmental regulation, which together may impose additional physiological constraints [11,17]. Consistent with these observations, clear physiological and transcriptional differences were observed between normal male/female and super-female sterlet in this study.

At the organismal level, super-female sterlet showed a markedly higher mortality rate than normal individuals, indicating reduced physiological robustness during juvenile development. This pattern agrees with earlier reports in gynogenetic sterlet [3,8]. Biochemically, super-female sterlet exhibited significantly elevated hepatic MDA levels, reflecting enhanced lipid peroxidation [18]. Although GSH-Px activity was also increased, suggesting an antioxidant response to oxidative stress [19], this response did not fully offset lipid peroxide accumulation, pointing to persistent oxidative imbalance.

Transcriptomic analysis further revealed pronounced changes in immune-related gene expression in super-female sterlet. Upregulation of genes involved in chemokine signaling, immune receptors, and cytokine pathways suggests substantial remodeling of immune-related transcription during development. In teleosts, immune signaling is closely linked to tissue remodeling and metabolic regulation during gonadal development [20,21]. In parallel, downregulation of genes associated with cell adhesion, protein folding, and lipid metabolism indicates impaired cellular homeostasis and membrane stability [22].

Taken together, the combined physiological and transcriptomic data indicate that super-female sterlet experience a state characterized by oxidative imbalance, altered immune-related regulation, and disrupted lipid metabolism, which may contribute to their reduced viability and higher mortality.

### 3.2. Potential Involvement of Ferroptosis Associated with Disrupted Arachidonic Acid Metabolism in the Reduced Viability of Super-Female Sterlet

Ferroptosis is a regulated form of cell death characterized by iron-dependent lipid peroxidation and has been increasingly reported in fish under conditions of metabolic disturbance, environmental stress, and immune challenge [23,24,25,26]. In this study, Transcripts associated with ferroptosis pathways were enriched in the gonadal transcriptome of WW-type super-female sterlet. Combined with the elevated lipid peroxidation and altered antioxidant responses observed at the physiological level, these findings indicate that lipid peroxidation-related cellular stress is associated with the reduced viability of WW-type individuals.

A key transcriptomic feature of WW-type super-females was the downregulation of genes involved in arachidonic acid metabolism, including PTGS2, PTGES, and PTGDS. Arachidonic acid is a major polyunsaturated fatty acid in cellular membranes and plays an important role in maintaining membrane integrity and regulating inflammatory signaling [27,28]. Reduced activity of arachidonic acid metabolic pathways may alter membrane lipid composition and limit the conversion of arachidonic acid into downstream lipid mediators.

Under such conditions, excess polyunsaturated fatty acids within cellular membranes may become more susceptible to oxidative damage, as lipid peroxidation of membrane PUFAs is a central feature of ferroptosis [29]. Consistent with this interpretation, WW-type super-females exhibited significantly elevated hepatic MDA levels, indicating enhanced lipid peroxidation at the physiological level. These changes suggest that disrupted arachidonic acid metabolism may contribute to increased lipid peroxide accumulation.

At the same time, GSH-Px activity was significantly increased in WW-type individuals, indicating an antioxidant response to elevated lipid peroxidation [30]. However, this response did not fully prevent oxidative damage, as reflected by persistently high MDA levels. In addition, downregulation of cysteine and methionine metabolism pathways may limit glutathione synthesis, which is required for effective GPX4-mediated detoxification of lipid peroxides [31,32,33]. Together, these changes indicate an imbalance between lipid peroxide production and antioxidant capacity.

Overall, the data suggest that altered arachidonic acid metabolism, enhanced lipid peroxidation, and constrained antioxidant defenses occur concurrently in WW-type super-female sterlet. Ferroptosis-related molecular signatures observed at the transcriptomic level may reflect increased cellular sensitivity to lipid peroxidation-mediated damage, providing a plausible explanation for the reduced viability of WW-type individuals.

## 4. Materials and Methods

### 4.1. Experimental Materials

F_2_ gynogenetic diploid juvenile sterlet were obtained from the Hulan Experimental Station of Heilongjiang River Fisheries Research Institute, Chinese Academy of Fishery Sciences. The experiment was conducted in the breeding workshop of the Heilongjiang River Fisheries Research Institute. The induction of gynogenetic diploids was performed following the method of Kinami et al. [8] with slight modifications. Semen collected from F_1_ sterlet males (~26 kg) was diluted with Hank’s balanced salt solution at a ratio of 1:9. One milliliter of the diluted semen was transferred into a sterile Φ90 mm Petri dish and subjected to UV irradiation (140 J/m^2^; 200 μW/cm^2^ for 70 s) with gentle shaking for 1 min. The inactivated sperm were collected into sterile self-sealing bags, filled with oxygen, protected from light, and stored at 4 °C. Sperm motility was assessed by activating the sperm with water and observing their activity under a microscope before use.

Sexually mature F_1_ females (~3 kg) were selected for egg collection. A total of 100 g of eggs was mixed with 10 mL of UV-irradiated sperm in 1000 mL of river water at 15 °C. After gentle stirring for 1 min, the eggs were transferred into a 20% talc suspension to de-adhesion for 5 min. The eggs were rinsed three times and incubated in river water at 15 °C. At 18 min post-fertilization, the eggs were subjected to thermal shock by immersion in 34 °C water for 2 min, followed by recovery at 15 °C. The treated eggs were then incubated under standard sturgeon embryo-rearing procedures. At 2 months post-hatch, gynogenetic diploids were genotyped as ZZ, ZW, or WW following the method of Kinami et al. [8].

### 4.2. Experimental Design and Sample Collection

A total of 120 healthy F_2_ gynogenetic diploid juveniles from each genotype (ZZ, ZW, WW), free of visible injuries and of similar size, were randomly assigned to fifteen indoor thermostatic recirculating tanks (60 cm × 40 cm × 50 cm). The average body weight of the fish was (5.74 ± 2.43) g, and the average body length was (8.68 ± 1.98) cm. The photoperiod was set to 12 h light:12 h dark. Water temperature was maintained at (19.0 ± 0.5) °C, with pH ~6.9 and dissolved oxygen ≥ 6 mg/L. Fish were fed twice daily at 7:00 and 19:00 at 3% of body weight (Shandong Shengsuo Feed Technology Co., Ltd., Yantai, China; crude protein ≥ 40%, crude lipid ≥ 10%, crude fiber ≤ 6%, crude ash ≤ 18%, moisture ≤ 12%). Prior to the morning feeding, waste removal and 50% daily water exchange were conducted. After a one-week acclimation, the formal experiment was initiated. Three experimental groups (ZZ, ZW, WW) were established, each with five replicates (24 fish per tank). Rearing conditions during the experiment were identical to those during acclimation.

Fish survival was monitored and recorded daily. On day 100, sample collection was performed. Four hours after morning feeding, one fish was randomly selected from each tank and anesthetized in an ice bath. Dissections were performed on a chilled clean board, and liver and gonad tissues were carefully excised using sterile tools. Samples were placed into sterile 1.5 mL microcentrifuge tubes, snap-frozen in liquid nitrogen, and stored at −80 °C.

### 4.3. Determination of Antioxidant Indices

Liver tissue from five individuals per genotype was homogenized in sterile saline (*w*:*v* = 1:9) on ice to prepare a 10% tissue homogenate. Three technical replicates were performed for each fish. The homogenate was centrifuged at 5000 rpm for 10 min, and the supernatant was used to measure superoxide dismutase (SOD), catalase (CAT), malondialdehyde (MDA), and glutathione peroxidase (GSH-Px). Protein concentration was determined using a commercial kit (Cat. No. A045-4-2, Nanjing Jiancheng Bioengineering Institute, Nanjing, China). SOD activity was measured using an SOD assay kit (Cat. No. A001-1, Nanjing Jiancheng Bioengineering Institute, Nanjing, China). CAT activity was measured using a CAT assay kit (Cat. No. A007-1, Nanjing Jiancheng Bioengineering Institute, Nanjing, China). MDA levels were measured using an MDA assay kit (Cat. No. A003-1-2, Nanjing Jiancheng Bioengineering Institute, Nanjing, China). GSH-Px activity was measured using a GSH-Px assay kit (Cat. No. A005-1, Nanjing Jiancheng Bioengineering Institute, Nanjing, China).

### 4.4. RNA Extraction, Library Construction, and De Novo Transcriptome Sequencing

Total RNA was extracted from gonadal tissue using TRIzol reagent. RNA concentration and purity were examined using a NanoDrop 2000 spectrophotometer (Thermo Fisher Scientific Inc., Waltham, MA, USA) and 1% agarose gel electrophoresis. Qualified RNA samples were sent to OE Biotech Co., Ltd. (Shanghai, China) for cDNA library construction and paired-end sequencing using the Illumina HiSeq 2500 platform (Illumina lnc., San Diego, CA, USA).

### 4.5. Quality Control, Assembly, and Functional Annotation

Raw reads were processed using Trimmomatic [34] to remove adaptor sequences, low-quality bases, and reads containing ambiguous nucleotides. Clean reads were assembled de novo using Trinity (version 2.4) [35] with the paired-end mode to obtain transcript sequences. For each gene cluster, the longest transcript was selected as the representative unigene. Unigene sequences were annotated against the Swiss-Prot database using BLASTx (v2.16.0, E-value < 1 × 10^−5^). Swiss-Prot IDs were subsequently mapped to the Gene Ontology (GO) database for functional annotation. KEGG pathway annotation was performed by aligning unigene sequences to the KEGG database.

### 4.6. Identification of Differentially Expressed Genes and Enrichment Analysis

Bowtie2 [36] was used to align reads to the reference genome, and read counts mapped to each unigene in each sample were calculated using eXpress (v1.5.1) [37]. FPKM (Fragments Per Kilobase of transcript per Million mapped reads) values were derived from the read counts provided by eXpress, which were used to estimate the expression levels of each unigene. The DESeq2 package in R (v4.4.1) was used for normalization (estimateSizeFactors) and differential expression analysis (nbinomTest) to obtain *p*-values and fold changes. Volcano plots were used to visualize all differentially expressed genes (DEGs), and genes with *p* < 0.05 and log_2_|Fold change| > 1 were further filtered for KEGG and GO enrichment analyses using the clusterProfiler package in R. The results were visualized with ggplot2, generating bar plots for GO enrichment and bubble plots for KEGG pathways.

### 4.7. Statistical Analysis

Data visualization was performed using ggplot2 in R, GraphPad Prism 9, and Microsoft Visio 2016. Survival rate data were analyzed using the log-rank test to compare survival curves between groups. For other statistical analyses, the Kruskal-Wallis test was applied using the kruskal.test function in R, and Dunn’s post-hoc multiple comparison test was performed using the dunn.test package in R.

## 5. Conclusions

This study combines physiological measurements with gonadal transcriptomic analyses to examine the reduced fitness of gynogenetic WW-type super-female sterlet. Super-female individuals exhibited persistent oxidative stress together with widespread changes in gene expression related to immune regulation and lipid metabolism. In particular, transcriptomic analyses revealed disrupted arachidonic acid metabolism, while enzymatic indicators reflected enhanced lipid peroxidation and altered antioxidant responses at the physiological level. Together, these findings indicate a close association between lipid metabolic disturbance and oxidative stress. In this context, the enrichment of ferroptosis-related genes may reflect increased cellular sensitivity to lipid peroxidation-mediated damage. Overall, these findings help explain the reduced viability of WW-type super-female sterlet and offer valuable insights for future efforts aimed at improving their robustness in all-female sturgeon breeding programs.

## Figures and Tables

**Table 1:** Quality statistics of the RNA-seq data.

Sample	Raw Reads (M)	Raw Bases (Gb)	Valid Bases (%)	GC (%)	Q20 (%)	Q30 (%)
ZZ-1	43.14	6.51	95.57	45.04	98.74	95.54
ZZ-2	39.21	5.92	95.59	44.64	98.71	95.49
ZZ-3	42.08	6.35	95.56	45.06	98.72	95.54
ZZ-4	36.89	5.57	95.57	45.49	98.77	95.58
ZZ-5	56.21	8.49	95.59	45.80	98.57	95.10
ZW-1	39.83	6.01	95.58	46.27	98.66	95.27
ZW-2	38.61	5.83	95.57	45.34	98.80	95.70
ZW-3	40.23	6.07	95.80	44.90	98.84	95.88
ZW-4	36.61	5.53	95.59	44.95	98.68	95.44
ZW-5	40.63	6.13	95.58	45.21	98.59	95.24
WW-1	41.70	6.30	95.58	44.84	98.72	95.44
WW-2	41.68	6.29	95.58	44.07	98.68	95.45
WW-3	46.92	7.09	96.55	44.54	98.71	95.48
WW-4	49.91	7.54	95.61	44.94	98.85	95.83
WW-5	40.20	6.07	95.58	44.59	98.72	95.54

## Data Availability

The data presented in this study are available on request from the corresponding author for scientific purposes.
